# Losing p53 loosens up ER-stress

**DOI:** 10.18632/aging.100847

**Published:** 2015-11-15

**Authors:** Sanguine Byun, Takushi Namba, Sam W. Lee

**Affiliations:** Cutaneous Biology Research Center, Massachusetts General Hospital and Harvard Medical School, Charlestown, MA 02129, USA

**Keywords:** p53, ER stress

The endoplasmic reticulum (ER) is the central intracellular organelle responsible for proper folding, localization, and post-translational modifications of proteins in eukaryotic cells. During conditions of cellular stress, incorrectly folded proteins can accumulate in the ER and trigger the unfolded protein response (UPR), which involves the activation of signal transduction pathways designed to restore correct protein folding and recovery from ER stress [1]. In rapidly dividing tumor cells, UPR can become constitutively activated, presumably because higher levels of proliferation require increased protein synthesis [1, 2]. In addition, the tumor micro-environment is often host to additional adverse conditions including nutrient and oxygen deprivation, leading to further perturbations of ER function. Therefore, it may come as no surprise that UPR has been shown to play a crucial cytoprotective role in tumor cells [2].

We have recently unveiled an unexpected role for the tumor suppressor p53 in regulating ER function via the IRE1α/XBP1 pathway [3]. IRE1α is an ER stress sensor that activates the UPR to restore ER function and maintain cellular homeostasis. It was observed that wild-type p53 suppresses IRE1α expression and activation, while the loss of active p53 potentiates the IRE1α/XBP1 pathway, enhancing ER function [3]. This finding suggests a survival benefit for cancer cells that can inactivate p53. Because cancer cells with high protein load on the ER would need enhanced ER function to cope with constant ER stress, losing p53 function would assist tumors to grow in adverse conditions by increasing IRE1α/XBP1 pathway activation. In addition, we found that a p53 target, CDIP1, acts as a proapototic signal mediator in conditions of ER stress [4]. The CDIP1/BAP31 complex was shown to transduce ER stress-mediated apoptotic signaling to the mitochondria and activate the intrinsic apoptosis pathway, suggesting that loss of p53 function may contribute to the survival of cancer cells by enhancing ER function as well as hindering ER stress-mediated apoptosis signaling.

The loss of p53 function in relation to ER stress is likely to have important consequences for the sustenance of cancer cell proliferation. Previous studies have demonstrated that the activation of p53 suppresses mTOR activity and consequently inhibits protein translation [5, 6]. The elimination of wild-type p53 function may therefore provide an opportunity for cancer cells to further promote unregulated proliferation, via elevated protein synthesis and a subsequent increase in ER function. We have revealed that wild-type p53 directly interacts with synoviolin (SYVN1) and can thereby stimulate SYVN1-dependent degradation of IRE1α. The loss of p53 disrupts SYVN1-mediated IRE1α downregulation, producing an ER stress-resistant phenotype by increased IRE1α/XBP1 pathway activity [3]. However, although the loss of p53 function facilitates enhanced ER function to manage high ER stress, this also generates an overreliance on IRE1α in cancer cells with p53 mutations. Indeed, we have found that pharmacological inhibition of IRE1α strongly suppresses protein secretion and potently induces cell death in such p53-deficient cancer cells [3]. Importantly, the IRE1α inhibitor exhibited significant inhibitory effects against p53-deficient human tumors in vivo compared to those with wild-type p53. These results illuminate an important proof-of-concept for targeting tumors with mutant p53 via inhibition of the IRE1α/XBP1 pathway.

Malignant transformation occurs through a combination of oncogenic pathway activation and the loss of tumor suppression. Understanding the consequences and identifying vulnerabilities caused by such changes can provide promising opportunities to selectively target cancer cells. Mutation in the p53 gene is the most frequent genetic alteration found in human cancer and inactivation of p53 is known as one of the strongest driving factors in cancer development and chemoresistance [7]. The new finding that loss of p53 activates the IRE1α/XBP1 pathway leading to enhanced ER function broadens our understanding of how cancer cells with mutant p53 survive, and also highlights a possibility for the development of novel therapeutic strategies.
